# Dual Targeting Topoisomerase/G-Quadruplex Agents in Cancer Therapy—An Overview

**DOI:** 10.3390/biomedicines10112932

**Published:** 2022-11-15

**Authors:** Silvia Salerno, Elisabetta Barresi, Emma Baglini, Valeria Poggetti, Sabrina Taliani, Federico Da Settimo

**Affiliations:** Department of Pharmacy, University of Pisa, Via Bonanno 6, 56126 Pisa, Italy

**Keywords:** topoisomerases, G-quadruplexes, telomeres, cancer, dual ligand, small molecules

## Abstract

Topoisomerase (Topo) inhibitors have long been known as clinically effective drugs, while G-quadruplex (G4)-targeting compounds are emerging as a promising new strategy to target tumor cells and could support personalized treatment approaches in the near future. G-quadruplex (G4) is a secondary four-stranded DNA helical structure constituted of guanine-rich nucleic acids, and its stabilization impairs telomere replication, triggering the activation of several protein factors at telomere levels, including Topos. Thus, the pharmacological intervention through the simultaneous G4 stabilization and Topos inhibition offers a new opportunity to achieve greater antiproliferative activity and circumvent cellular insensitivity and resistance. In this line, dual ligands targeting both Topos and G4 emerge as innovative, efficient agents in cancer therapy. Although the research in this field is still limited, to date, some chemotypes have been identified, showing this dual activity and an interesting pharmacological profile. This paper reviews the available literature on dual Topo inhibitors/G4 stabilizing agents, with particular attention to the structure–activity relationship studies correlating the dual activity with the cytotoxic activity.

## 1. Introduction

Cancer is a serious disease that, despite the increasingly widespread prevention campaign and advances in therapeutic treatments, still remains a major challenge for human health around the world [1]. At the molecular level, cancer is considered a genetic disease whose development is strictly related to the aberrant expression of tumor silencers and oncogenes [2].

To date, it is widely accepted that the crucial event which limits the lifespan of normal cells is the progressive erosion of the extremities of chromosomes with specialized sequences, termed telomeres [3]. Telomeres include tandem repetitions of DNA sequences and, in particular, human telomeres are constituted by repeats of the hexanucleotide sequence 5′-TTAGGG in double-stranded DNA, being one strand rich in guanine (G strand) and the complementary one in cytosine (C-strand) [4].

The primary function of telomeres is to plug the ends of the chromosome to maintain its stability [5]. To satisfy the capping function, telomeres must have a minimum length to form three-dimensional structures named T-loops [6]. In addition, to protect the end of chromosomes, telomeres are involved in further important functions associated with the cell cycle, replication and aging [3,7]. During aging, most human tissues and organs undergo a telomere shortening, and each cycle of cell division causes a shortening of the telomeres by about 50–200 nucleotides [6,8]. To compensate for this telomere shortening, the enzyme telomerase, a DNA polymerase, adds telomere repeat sequences on the ends of telomeres, ensuring the telomere has the correct length for the subsequent cell division cycles [9].

As cancer incidence abruptly increases during aging, cancer can be viewed as an aging-associated disease in which telomere shortening might play a significant role. Initially, telomere shortening was proposed as a tumor suppressor mechanism, being a limiting factor in the lifespan of human cells [3]. Accordingly, cancer cells need to stabilize telomeres in order to gain immortal proliferative capacity. Although more than 90% of human cancers (liver, lung, breast, prostate, colon, brain, pancreas and head and neck cancers, as well as malignancies of the hematopoietic system) show a strong reactivation of telomerase [10], they have very short telomeres, much shorter than the surrounding healthy tissue [10]. A plausible explanation for this apparent paradox derives from studies in telomerase-deficient mice, showing that telomere dysfunction increases the rate of cancer initiation by inducing chromosomal instability and a DNA damage response [11,12]. According to these studies, telomere shortening seems to have a dual role in cancer. It can increase tumors’ initiation by inducing chromosomal instability and genetic alterations that lead to cellular transformation. However, tumor cells need to stabilize telomere shortening to avoid too high levels of instability, which would ultimately lead to the cancer cells’ death; thus, they still reactivate telomerase [13] to maintain telomeres at a constant length [3].

Based on these observations, human telomerase has been proposed as a new and highly selective target for antitumor drug design campaigns [9,14,15]. Sen and Gilbert suggested that telomere DNA sequences may join to initiate the alignment of four sister chromatids to form parallel guanine quadruplexes called G-quadruplexes (G4). This hypothesis was subsequently confirmed by biophysical studies on DNA oligonucleotides with sequences similar to those from telomeres that highlighted the stable formation of G4 structures under near-physiological conditions in vitro [16]. Specifically, the protruding 3′ single-strand of the telomere DNA, more thermodynamically stable than double-stranded DNA, can adopt the peculiar G4 fold, which is a secondary four-stranded DNA helical structure composed of guanine-rich nucleic acids [17,18]. About 50% of human genes are expected to produce G4, which is generally found close to oncogenic promoters rather than to the neighboring household genes [19].

**Figure 1:**
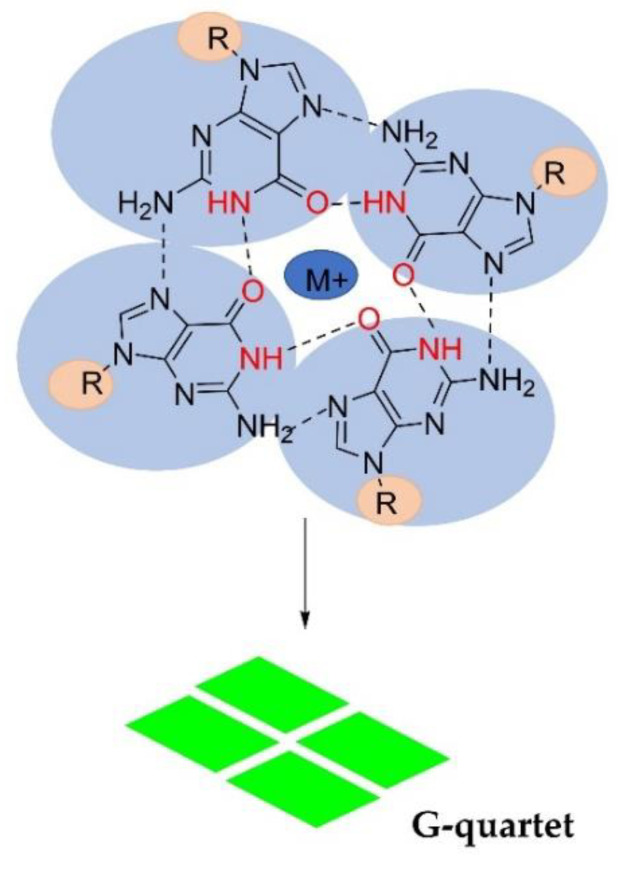
G-Quadruplexes framework: structure of a G-quartet consisting of a coplanar structure of four guanines bound with their N1 and O6 (outlined in red) and stabilized by a central monovalent metal cation (M^+^).

G4 typically consists of three or even more layers of G-quartet, each of which is formed by four planar guanines linked by hydrogen bonds and stabilized in the center by a monovalent cation (more frequently K^+^) (Figure 1) [20,21,22].

Changes in G4 formation/stability can alter telomerase activity [23,24] and transcription efficiency (inhibiting or promoting it) [25], stall DNA replication and induce genome instability [26]. These changes can be triggered chemically with G4-ligands or by proteins that modulate G4 formation. G4 ligands able to modulate or stabilize G4 structures, and consequently block cellular replication or oncogenes’ expression, have, therefore, the potential to be used in anti-tumor treatment strategies [27].

Formation and/or stabilization of G4 structures represent potential therapeutic tools against tumor cells [27,28], and three main therapeutic strategies have been investigated [29]. The first is based on the observation that most promoters of oncogenes harbor more G4 motifs than the promoters of regulatory or tumor suppressor genes [30], and changes within the G4 structure in the promoters correlate with a reduction in gene expression (e.g., MYC [31], VEGF [32], BCL2 [33], KRAS [34] and KIT [35]). Thus, regulating oncogene expression by inducing G4 structures in the corresponding promoters might represent a valuable strategy to reduce tumor growth. As an example, inducing G4 structures within the oncogene promoter MYC [31,36,37,38] blocks the expression of MYC, a transcription factor that is upregulated in 70% of cancers, altering cell proliferation, metabolism and immune evasion [37]. Several G4 ligands are reported to reduce tumor growth as a consequence of a decreased expression of MYC and other oncogenes [39], even if, due to the low selectivity of these compounds, other molecular mechanisms cannot be excluded [40]. The second therapeutic strategy is based on the evidence that, under specific conditions, G4 structures can cause genome instability [20], inducing genetic alterations (e.g., point mutations, insertion, deletion, telomere addition, or even epigenetic changes), which are observed in many cancers. Treatment with G4 ligands might increase genome instability leading to enhanced DNA double-strand breaks, replication pauses, micronuclei formation and telomere maintenance problems [41,42]. Finally, G4 formation/stabilization at the telomeres was used to block telomerase activity in tumors, thus hindering uncontrolled DNA replication. In fact, telomerase activity is upregulated in about 85–90% of cancer cells, which are thus able to replicate without telomere shortening [43]. The G4 structures at the telomeres can alter telomerase binding and block telomerase activity in tumor cells [44], while somatic cells do not express telomerase and are thus not affected.

**Scheme 1:**
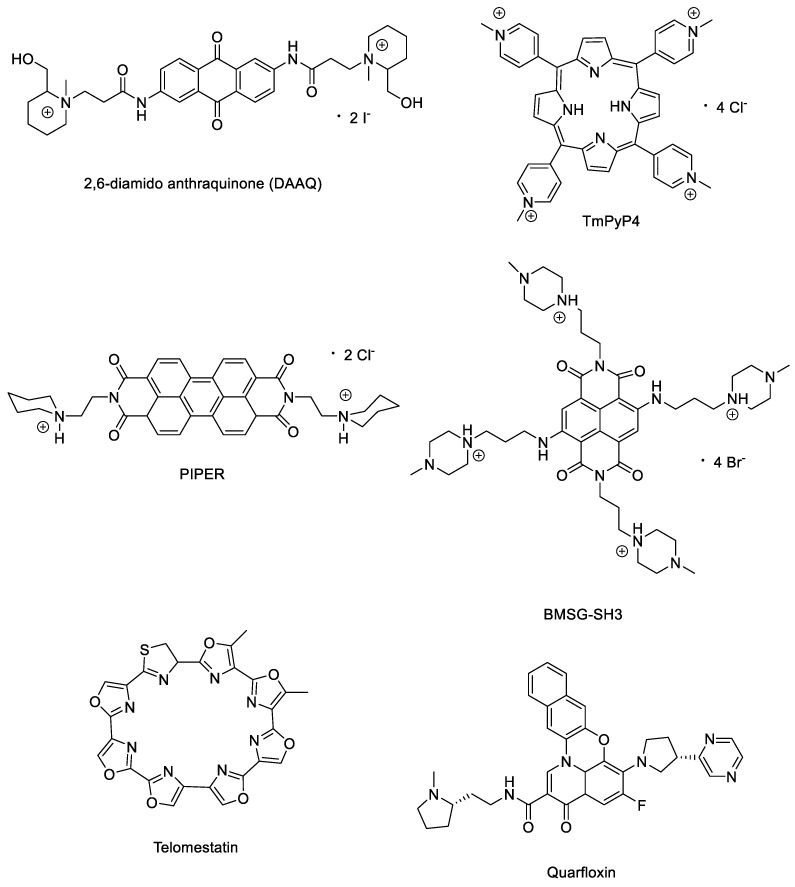
Chemical structures of some interesting G−quadruplex ligands.

Based on these considerations, small molecules stabilizing or inducing G4 structures have been suggested as potential anticancer drugs [45,46]. In fact, a number of interactive G4 compounds not only inhibit telomerase activity in cell-free systems and in vitro, but they also affect telomere shortening and, above all, cell death in cancer cells [47,48,49,50,51]. To date, numerous G-quadruplex ligands are listed in the G4 ligands database (G4LDB). As examples, 2,6-diamidoanthraquinone (DAAQ) [45], TmPyP4 [52], and PIPER [53] (Scheme 1) were the first described G-quadruplex interactive agents, and they were used as lead compounds for the development of many other G4 interactive analogs. In particular, TmPyP4 is commercially available, and it has been widely used as a reference standard for biological assays. Structural modifications to improve the physicochemical properties of PIPER led to the development of naphthalene diimide derivatives [54], and, among them, BMSG-SH-3 (Scheme 1) entered clinical trials. BMSG-SH-3, together with telomestatin (Scheme 1) [55], a natural macrocyclic compound isolated from *Streptomyces anulates*, have been shown to promote cell cycle arrest and apoptosis in glioblastoma and in uterus, prostate and gastrointestinal cancers. To date, quarfloxin (Scheme 1), an anti-neoplastic fluoroquinolone derivative developed by Cylene Pharmaceuticals, is the only G-quadruplex ligand to have entered phase II clinical trials (ClinicalTrials.gov identifier: NCT00780663), due to its ability to interact with G4s in vivo [56]. Ultimately, quarfloxin was discarded due to its poor bioavailability.

G4 stabilization impairs telomere replication and triggers the specific involvement and activation of several protein factors at telomere levels, such as topoisomerases (Topos) and poly-(ADP-ribose) polymerases (PARPs) [57,58]. In addition, in cancer cells, G4 can be converted back into duplex DNA, thanks to the action of highly expressed Topo enzymes [59], thus activating c-MYC transcription [60].

**Scheme 2:**
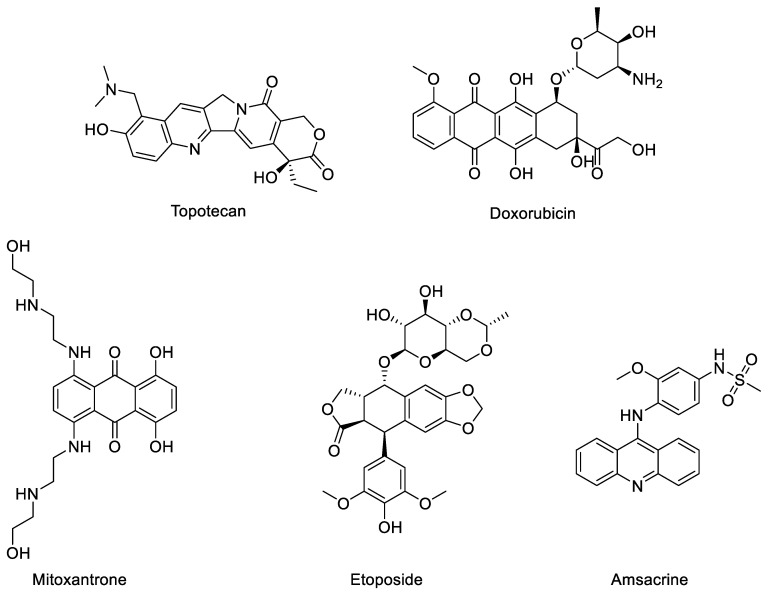
Chemical structures of some marketed Topoisomerase inhibitors.

DNA Topos are ubiquitous nuclear enzymes involved in solving all the topological problems of the DNA during all cellular transactions [61]. In eukaryotic organisms, Topos can be classified into two main different classes: topoisomerase I (TopoI) and topoisomerase II (TopoII), with different structural organization, enzymatic catalysis modalities and biological functions. Under physiological conditions, Topos operate transient DNA cleavages as the relegation phase is faster than the breaking one and, therefore, is well tolerated by cells. On the other hand, if the amount or duration of the breakage becomes too high, the DNA undergoes permanent changes, which prevent the progression of the subsequent phases, and this is of fundamental importance in cancer cells, which are in continuous proliferation [62,63,64]. Topo inhibitors can carry out their activity by exploiting two different molecular mechanisms. The first consists of the formation of a blocked ternary Topo-DNA-inhibitor cleavable complex [65,66,67,68], the accumulation of which prevents the enzyme from completing its catalytic cycle; DNA breaks are stabilized, thus leading to a cytotoxic effect. The enzyme is therefore covalently trapped in the DNA, and such a cleavable complex is termed “poisoned”. Therefore, the inhibitors that act with this mechanism are termed Topo poisons [68,69]. The second one consists of interfering with the binding of Topos to DNA or in the catalytic inhibition of the Topos, stopping DNA transcription and replication that ultimately leads to cell death. Compounds that act by this mechanism are called Topo catalytic inhibitors or suppressors and may belong to different classes [65,70,71]: (i) DNA binders or intercalators, which change the shape of free DNA so that Topos bind less effectively [71]; (ii) agents that bind the free enzyme (such as some porphyrin compounds) and prevent the nicking reaction [72]; (iii) agents that react with specific amino acids of Topos (such as cysteines) and inhibit the enzyme [73]; (iv) molecules that compete for the ATP binding site of TopoII, resulting in the catalytic inhibition of TopoII [65,70]; and (v) compounds that can bind to the DNA–Topos complexes and prevent cleavage [74]. The ability to interfere with the Topos’ activity has been exploited as an effective strategy for cancer therapy and, in fact, many Topo (I or II) inhibitors have been marketed drugs for several decades [75,76,77,78,79,80,81]. Marketed Topo inhibitors for the treatment of cancer include both TopoI and TopoII poisons. The class of TopoI poisons includes camptothecins such as topotecan (Scheme 2), approved in 1996 for the treatment of metastatic ovarian cancer and, later, in 1998 for the treatment of small cell lung cancer. Anthracyclines (e.g., doxorubicin), anthracenediones (i.e., mitoxantrone), epipodophyllotoxines (e.g., etoposide) and amsacrine (Scheme 2) are TopoII poisons used in many types of cancer including breast, lung, ovarian cancer and also acute leukemia [63]. Despite their clinical effectiveness, the use of these drugs is limited by several important side effects. Their mechanism of action appears to be responsible for further toxicities, including cardiotoxicity (especially anthracyclines) and secondary leukemia [82,83,84]. Moreover, multi-drug resistance (MDR) has been one of the challenges in targeting cancer cells employing TopoI or TopoII inhibitors. This acquired resistance may be due to reduced drug absorption, conformational changes, overproduction of the target enzyme and reduced activation and/or increased pharmacological catabolism [85,86].

**Figure 2:**
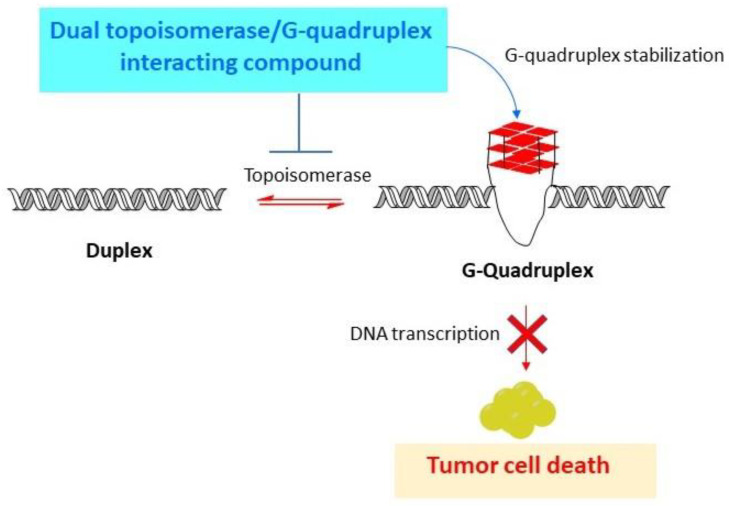
Rationale for developing dual-targeting topoisomerase/G-quadruplex agents in cancer therapy.

A new opportunity for pharmacological intervention to achieve greater antiproliferative activity, circumvent cellular insensitivity and resistance and decrease undesirable side effects, might be offered by the concomitant G4 stabilization and inhibition of Topos (Figure 2) [87].

The present review analyzes the available literature on dual Topo inhibitor/G4 stabilizing agents, with particular attention to the structure–activity relationship (SAR) studies. The final goal is to highlight the structural requirements necessary for the development of potent dual modulators of these targets, thus providing useful data to the scientific community involved in the development of more efficient and safer anticancer agents.

## 2. Dual Topoisomerase Inhibitor/G-Quadruplex Interacting Agents

While Topos’ inhibitors have long been widely studied, and some of them have entered the market for the treatment of cancer, and G4-interacting agents have been studied for years, some of them entering the clinical trial phase, the dual topoisomerase inhibitor/G-quadruplex interacting agents are, instead, still poorly studied. Indeed the combination of a TopoI inhibitor with RHPS4 (3,11-difluoro-6,8,13-trimethyl-8H-quino[4,3,2-kl]acridinium methosulfate), a G4 stabilizing agent, was found to be a highly effective treatment, allowing for complete tumor regression and a significant increase in the overall survival of mice, even when the treatments are initiated at a very advanced stage of tumor growth [58]. For this reason, there is still much to be investigated and documented in this field.

In practice, single-target treatment in severe and complex diseases involving many pathogenic factors such as, for example, cancer, may be inadequate due to the activation of compensatory mechanisms and alternative pathways [88,89]. As a result, the use of a drug simultaneously targeting different pathways involved in the onset and/or progression of the tumor can lead to a synergistic effect that might have considerable potential in anticancer therapy. The development of G-quadruplex agents, for which MDR has not yet been recognized and that simultaneously inhibit Topos activity, might also minimize drug resistance, because cancer cells are frequently incapable of adapting to the simultaneous toxic effects of dual-targeting agents. In addition, the enhanced therapeutic effects of a multitarget drug require lower doses, with the potential to reduce side effects compared to individual drug treatments [87,90].

In this manuscript, dual Topo inhibitor/G-Quadruplex interacting agents reported in the recent literature are described and classified, focusing attention on their chemical structures. In particular, the biological profiles of the reported compounds are discussed, with particular attention to those able to exert cytotoxicity in vitro towards some human tumor cell lines and also in vivo (if carried out). When possible, SARs are also discussed.

### 2.1. Fluoroquinoanthroxazines

**Scheme 3:**
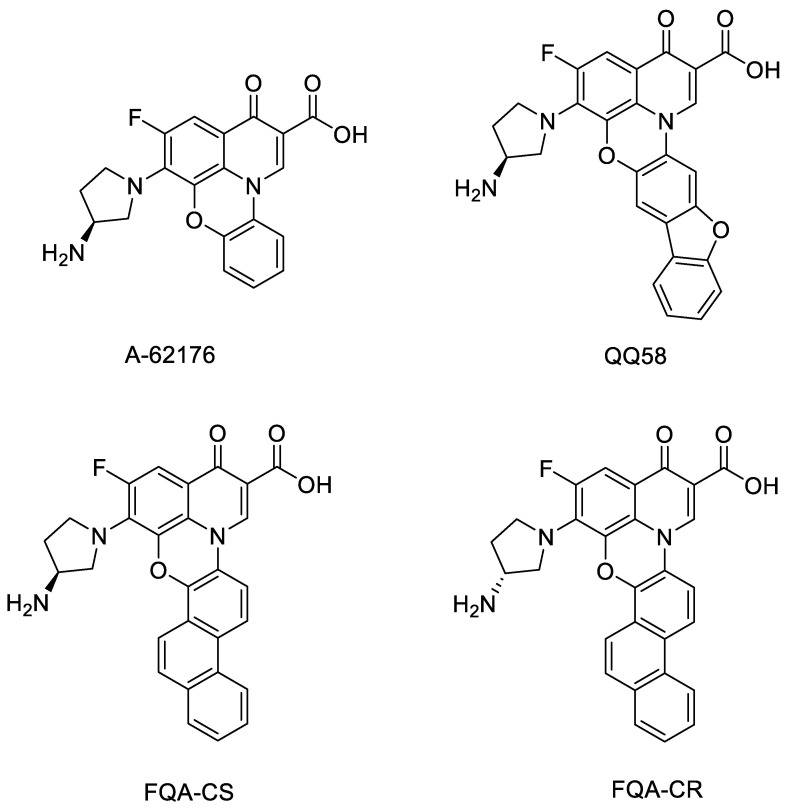
Chemical structures of fluoroquinolone analogs and FQAs.

Fluoroquinolones (e.g., norfloxacin) are well-known antimicrobial agents that inhibit bacterial DNA gyrase [91], but, recently, fluoroquinolones have been demonstrated to be Topo II [92], or telomerase inhibitors [93]. Among them, A-62176, a quinobenzoxazine, (Scheme 3), showed good activity against several human and murine cancer cell lines in vitro and in vivo [94], acting as a catalytic inhibitor of Topo II [95], or as Topo II poison under certain conditions [96]. Thus, it was used as a lead compound for designing many potent Topo II inhibitors by means of a structure-based approach [97].

The extended aromatic conjugation system of A-62176 suggests that quinobenzoxazines may also intercalate with the more expansive G-quadruplex DNA system and thus act as telomerase inhibitors [47,97].

Initially, A-62176 was used as starting point to design the extended analog QQ58, a fluoroquinophenoxazine (Scheme 3) [47], in which the phenoxazine ring selectively enhances the stacking interactions with G-quadruplex structures, thereby increasing the telomerase inhibition. At any rate, the Topo II poisoning activity of the parent compound A-62176 was lost in the QQ58.

So, in 2003, Kim et al. [98], with the aim of identifying new compounds with increased G-quadruplex interactions, while maintaining the TopoII poisoning effects, designed a series of fluoroquinoanthroxazines (FQAs) [47,97]. A naphthyl extension was inserted in the FQAs to increase the planar aromatic system with respect to the parent compound A-62176, in which the π-π stacking interactions were considered insufficient to adequately stabilize the G-quadruplex.

The polymerase inhibition and DNA cleavage assays, carried out on the FQAs, gave important indications of the interaction with G-quadruplex and of TopoII poisoning activity, respectively. Among the newly synthesized FQAs, two compounds, namely FQA-CS and FQA-CR (Scheme 3), showed good cytotoxicity (1.1 μM and 0.46 μM, respectively) towards MCF7 breast cancer cells, due to a dual mechanism of action towards the TopoII and G-quadruplex (Table 1) [98].

In particular, while A-62176 is both a TopoII poison and a catalytic inhibitor, the FQAs were found to be only TopoII poisons, with FQA-CS being by far the most potent one and for which the DNA cleavage activity increased in a concentration-dependent manner [98].

**Figure 3:**
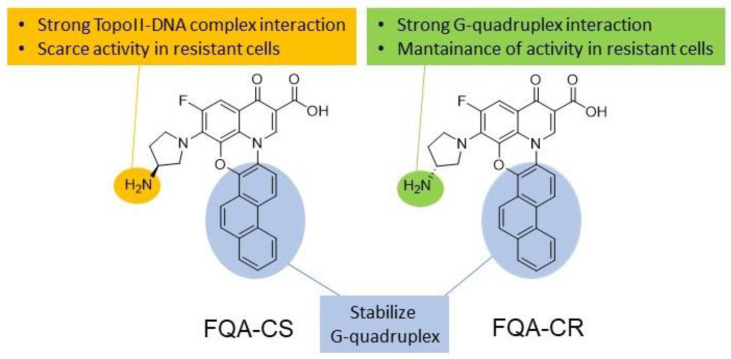
Graphical representation of the fluoroquinoanthroxazines’ functional groups necessary for the interaction with the target proteins.

Moreover, the R-enantiomer (FQA-CR) interacts with G-quadruplexes to a greater extent than the S-enantiomer (FQA-CS) does. This is due to the amino hydrogens of the aminopyrrolidine in the FQA-CR that are more favorably disposed than the corresponding ones in the S-enantiomer, so facilitating the formation of a stabilizing hydrogen bonding interaction with the 5′-phosphate group of G-quadruplexes [47]. In contrast, FQA-CS is more favored for binding with TopoII-DNA complexes [96]. FQA-CS activity significantly decreases in resistant cells, while FQA-CR retains a similar level of potency against the TopoII-resistant cells compared to the TopoII-sensitive cells, Figure 3. This might represent an important advantage in the development of drugs with a dual mechanism of action for treating tumors having developed resistance mechanisms for one specific target.

### 2.2. Indenoisoquinolines

Indenoisoquinolines are largely reported as TopoI inhibitors, with improved physicochemical and biological properties compared to the classical camptothecin TopoI inhibitors. They are clinically used for the treatment of various solid tumors [99,100,101,102], and some of them have entered phase I clinical trials for relapsed solid tumors and lymphomas [103,104,105].

**Scheme 4:**
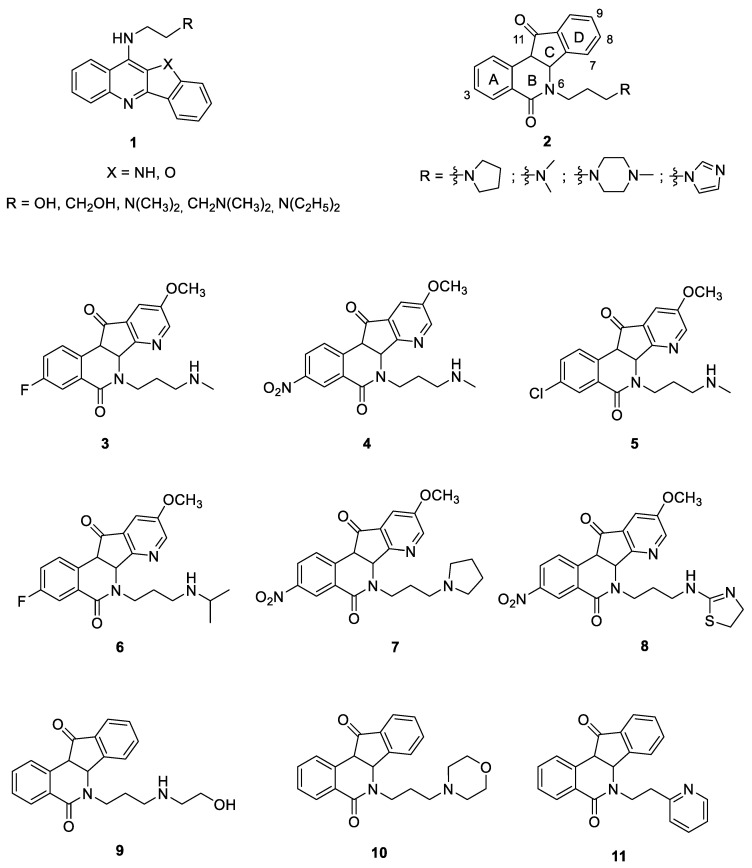
Chemical structures of quindolines (**1**) and indenoisoquinolines **2**–**11** (**2** general structure**, 3**–**11** representative compounds).

Based on a report by Ou et al. suggesting that monosubstituted quindoline derivatives (general formula **1**, Scheme 4) show a high stabilization of G-quadruplex [106], a series of tetracyclic 6-substituted indenoisoquinolines **2,** featuring only one side chain, was designed (Scheme 4) [107]. Indeed, the indenoisoquinolines **2** are represented by a crescent shape and share a structural similarity with the quindoline **1** compounds.

The compounds of series **2** resulted in a new class of G-quadruplex-stabilizing agents, with good antiproliferative activity in vitro on gastrointestinal stromal tumors (GIST882) and on colon adenocarcinoma (HT-29) cell lines, with IC_50_ values ranging from 0.3 to 23.0 μM, with HT-29 being the most sensitive cell line for all the derivatives **2**.

On the basis of these premises, in 2019, 56 indenoisoquinoline derivatives were examined and 44 of them were proven to stabilize G-quadruplex by fluorescence resonance energy transfer (FRET) melting experiments [108]. Moreover, Western blotting experiments, using MCF-7 breast cancer cells, were carried out to assess the MYC-inhibitory effects of the 44 indenoisoquinolines, which were then ranked into four groups, i.e., strong, medium, weak and no inhibition. Interestingly, the most potent G4-stabilizing derivatives (apparent binding affinity K_d_ values ranging from 5.6 to 23.9 nM, Table 2) also showed a potent MYC-inhibitory effect. All 44 compounds, for which the TopoI inhibitory activity has already been previously determined [102,109,110,111,112,113,114], were also tested for their antiproliferative activity on the NCI-60 cancer cell lines screen [115,116], and 31 of them showed potent antiproliferative activity (some mean graph midpoint, MGM and values are reported in Table 2), often related to TopoI inhibition or G-quadruplex stabilization activity [108].

The most interesting results concerning indenoisoquinolines are shown in Table 2; an analysis of these results with regard to the compounds’ ability to interact with G-quadruplex allowed us to identify clear SARs.

In particular, the 7-azaindenoisoquinolines **3**–**8** (Scheme 4) showed high selectivity for G-quadruplex, while compounds with an N6-substitution, but no A- or D-ring substituents, were less selective. As the indenoisoquinolines lacking the aminopropyl side chain (structures not reported) were found to be poor MYC-binders and G4-stabilizers, the N6-alkyl amine side chain proved to be important for G-quadruplex binding and stabilization, due to the ability of the positively charged basic N to engage favorable electrostatic interactions with the negatively charged phosphate backbone in the G-quadruplex groove.

**Figure 4:**
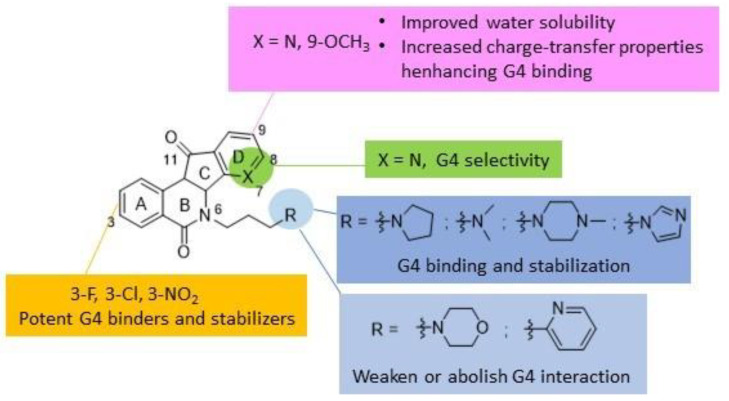
Graphical representation of the indenoisoquinolines’ functional groups necessary for the interaction with the target proteins.

In fact, the 9-methoxy-7-azaindenoisoquinolines (**3**–**8**, Scheme 4) developed to improve water solubility and to increase the charge-transfer properties [117,118], strongly bound G-quadruplex, and those with substituents, such as fluoro- (**3**, **6**, Scheme 4), nitro- (**4**, **7**, **8**, Scheme 4) and chloro- (**5**, Scheme 4) at position 3 of the A ring, were potent G-quadruplex binders and stabilizers, Figure 4.

Even in compound **9**, although without substituents on the A and D rings, a strong interaction with the G-quadruplex is maintained, again emphasizing the importance of the N6-aminoalkyl side chain for a favorable interaction. The presence of bulkier N-containing ring systems can sterically hinder the binding. For example, the presence of a more cluttered ring containing N (compound **10**), and a ring with a reduced positive charge for N (compound **11**), weakened and abolished the G-quadruplex interaction, respectively (Table 2).

### 2.3. Dibenzoquinoxalines

Quinoxaline moiety has gained considerable attention in the field of contemporary medicinal chemistry, thanks to its documented wide range of pharmacological, including anticancer, activities [119].

**Scheme 5:**
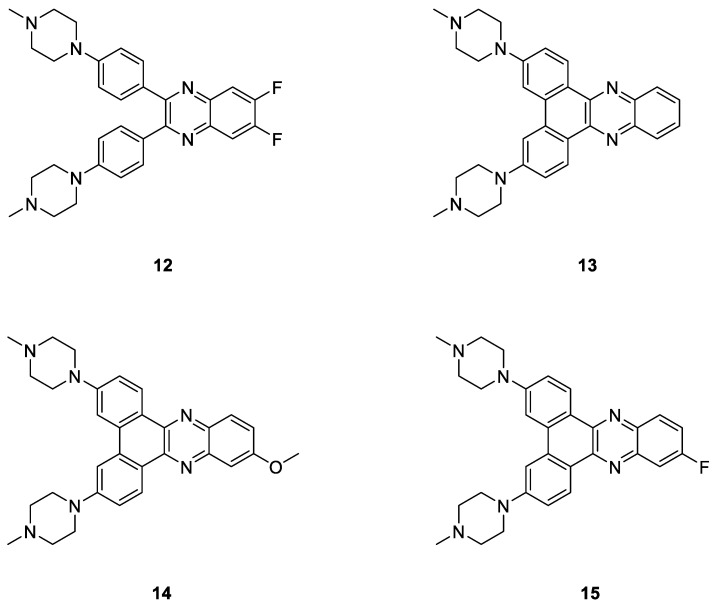
Chemical structures of dibenzoquinoxalines **12**–**15**.

A substituted quinoxaline derivative **12** (Scheme 5), inhibiting triple-negative breast cancer (TNBC) growth thanks to its G-quadruplex binding, was recently reported by Hu et al. [120]. Although quinoxalines retain suitable structural requirements to target Topos [119], compound **12** showed little TopoI inhibitory activity. Therefore, in 2021, it was suitably modified with the aim to obtain dual ligands, targeting both Topo I and the G-quadruplex with high inhibitory effects on tumor growth [121].

Exploiting the Topo inhibitors’ structures, which are always characterized by a coplanar skeleton (e.g., topotecan, doxorubicin and etoposide) [122], compound **12** was modified by extending its aromatic core by bridging the two pendant phenyl groups at positions 2 and 3.

In this context, a new series of 12 dibenzoquinoxaline derivatives was obtained, and, among them, compounds **13**–**15** (Scheme 5) were potent TopoI and c-MYC transcription inhibitors, also inhibiting cancer cell growth in TNBC cell lines, with IC_50_ values in the range of 1.0 μM (Table 3). These results suggested that there was a combined effect of TopoI inhibition and c-MYC inhibition on the antitumoral activity of the tested compounds.

**Figure 5:**
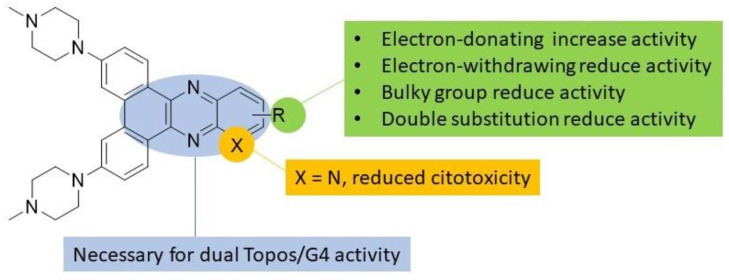
Graphical representation of the dibenzoquinoxalines’ functional groups necessary for the interaction with the target proteins.

For this series of dibenzoquinoxaline derivatives, it was possible to define some SAR. The quinoxaline core was an important prerequisite, as extending or altering this structure weakened the TopoI or c-MYC transcription inhibitory activity. The insertion of a nitrogen atom to obtain the pyrido [2,3-*b*]pyrazine scaffold (structure not reported) abolished the inhibitory activity on the c-MYC transcription and greatly reduced the cytotoxicity; this is probably due to the increase in molecular polarity, which may reduce the compounds’ ability to penetrate cellular membranes and reach their targets in the nuclei, thus resulting in poor intracellular activity. Regarding the substituents inserted into the quinoxaline core, an electron-donating substituent (−OCH_3_) increased the overall activity, while an electron-withdrawing one (−CF_3_) reduced it. Moreover, a bulky group in the same position, regardless of its electronic characteristics, seems to reduce the activity of the derivatives, probably due to a weaker interaction with the target. However, the introduction of two substituents decreased the overall activity, indicating that the extra substituent may sterically hinder the binding, Figure 5.

Among all the dibenzoquinoxaline derivatives, compound **14** was selected for further studies, and, finally, it was identified as the most promising dual ligand, effectively inhibiting TopoI activity and strongly stabilizing the G-quadruplex, so inhibiting the c-MYC. In particular, RT-PCR, Western blotting and other biological assays revealed that compound **14** is an effective non-intercalative TopoI catalytic inhibitor, and it strongly binds and stabilizes the G-quadruplex. Moreover, cell-based assays demonstrated the ability of compound **14** to inhibit cancer cell growth by inducing apoptosis. It also showed good in vivo antitumor activity in an MDA-MB-231 tumor-bearing mouse model, which is a reliable animal model for human triple-negative breast cancer (TNBC) [121].

Taken together, these results were extremely encouraging, as they affirmed compound **14** as a sound and viable candidate for the development of new dual Topo1 and G4 ligands.

### 2.4. Ruthenium(II) Polypyridyl Complexes

Ru(II) complexes with polypyridyl ligands have become prominent DNA-intercalating agents and have been widely investigated, thanks to a combination of easily constructed three-dimensional spacial structures and abundant photophysical properties [123].

**Scheme 6:**
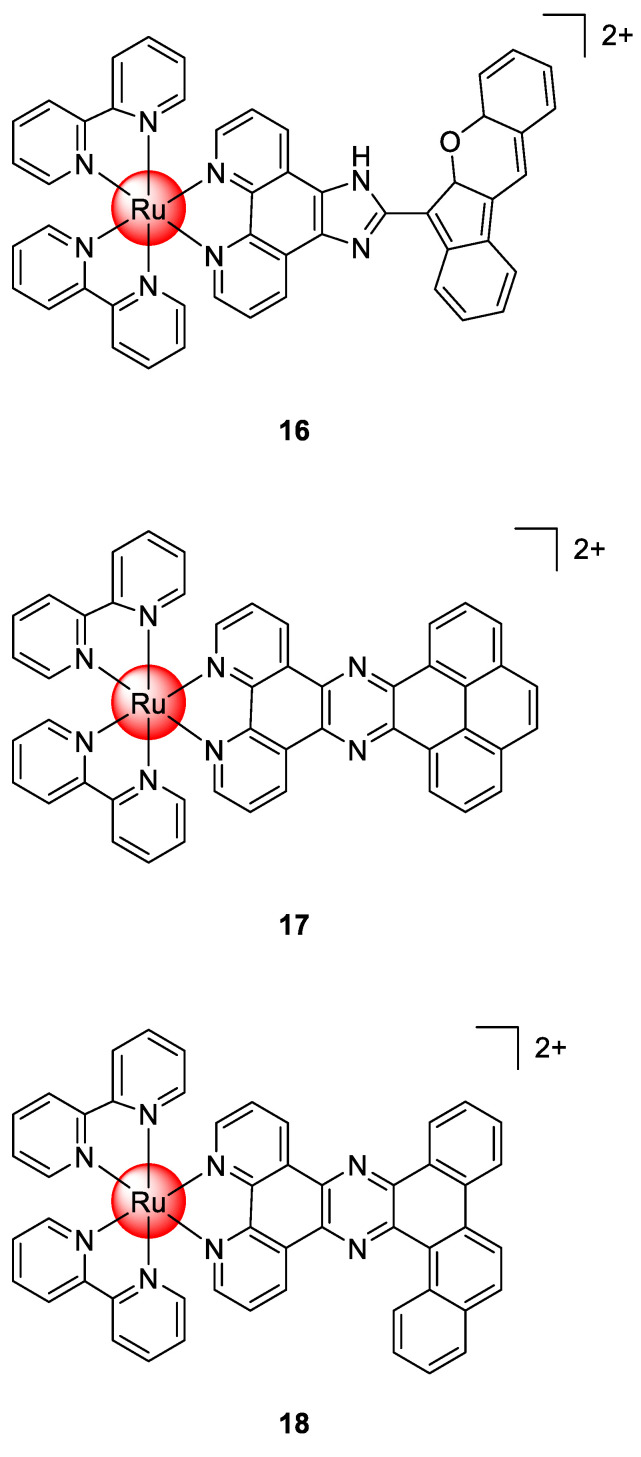
Chemical structures of ruthenium(II) polypyridyl complexes **16**–**18**.

In 2015, [124] three ruthenium polypyridyl complexes [Ru(bpy)_2_(icip)]^2+^ (**16**), [Ru(bpy)_2_(pdppz)]^2+^ (**17**) and [Ru(bpy)_2_(tactp)]^2+^ (**18**) (Scheme 6, bpy = 2,2′-bipyridine, icip = 2-(indeno[2,1-*b*]chromen-6-yl)-1*H*-imidazo-[4,5-*f*][1,10]phenanthroline, pdppz = phenanthro[4,5-*abc*]dipyrido[3,2-*h*:2′,3′-*j*]phenazine and tactp = 4,5,9,18-tetraazachryseno[9,10-*b*]-triphenylene) were investigated [125,126].

Ru-complexes **16**–**18** interact with the G-quadruplex according to two different mechanisms, which consist of a stacking mode for complexes **17** and **18** and an intercalation mode for complex **16**, in which the rotatable C–C bond allows the ligand to be able to assume the right conformation to insert itself among the DNA quartets. Behind the G4 interaction, derivative **16** was also a TopoI poison, while complexes **17** and **18** were dual TopoI/TopoII poisons.

The MTT assay after 48 h of drug treatment revealed that complexes **17** and **18** are able to exert acute cytotoxicity at a similar concentration of cisplatin, whereas derivative **16** shows to be a weaker cytotoxic complex. These results were in accordance with the ability of compounds **16**–**18** to inhibit Topos (Table 4) and to induce apoptosis. Moreover, a cell cycle analysis indicated that, although compounds **16**–**18** do not exactly follow the same anticancer mechanism, they can all induce cell apoptosis.

## 3. Conclusions and Future Perspective

Telomere biology has greatly evolved over the past 70 years: it started with McClintock’s observation that chromosomes need protection and was followed by the 2009 Nobel Prize in Physiology and Medicine to Elizabeth Blackburn, Carol Greider and Jack Szostak for the discovery of telomerase and the effects of telomere shortening on cells. Telomere function has been implicated in the replicative aging process and shown to play a major role in the establishment of genome instability in cancer development.

The main function of telomeres is to plug the chromosomal ends to maintain chromosomal stability and, to fulfill the capping function, the telomeres must have a minimum length and form three-dimensional structures (T-loops).

One of the mechanisms that leads to “telomere uncapping”, which is an alteration of the T-loop structure, is provided by the G-quadruplex (G4) stabilization. In this view, G4 stabilization is widely recognized as an interesting target to block cellular replication and/or expression of oncogenes, including MYC, and the small molecules able to modulate or stabilize the G4 structures can represent useful tools for the development of effective antitumor therapeutic approaches. However, the topoisomerases (Topos) can dissipate this negative supercoiling, leading to the continuous activation of the transcription of several oncogenes, such as MYC.

In this context, the dual targeting of Topos and G4 appears to be an innovative and promising strategy for the development of effective anticancer drugs.

To date, the dual Topo inhibitor/G-quadruplex interacting agents are still poorly studied, and the few classes reported in the literature are related to fluoroquinoanthroxazines [98], indenoisoquinolines [108], dibenzoquinoxalines [120] and ruthenium(II) polypyridyl complexes [124]. For these classes, a focus on SAR studies, the biological profile as well as the reported ability to exert cytotoxicity against tumor cell lines in vitro and in vivo (if carried out), were described.

Although the research in the field of dual Topo inhibitor/G-quadruplex interacting agents is still limited, some compounds possess very interesting pharmacological profiles, highlighting some chemotypes as promising scaffolds for dual G4 and Topos activity. In particular, the dibenzoquinoxaline derivative **14** [120] was one of the most promising dual ligands, effectively inhibiting TopoI activity and strongly stabilizing the G-quadruplex; it was also shown to inhibit the proliferation of the triple-negative breast cancer growth in in vivo studies.

In conclusion, the purpose of this review was to highlight to medicinal chemists how targeting both Topos and G4s may represent a valuable option within drug discovery programs to develop antitumor agents that are increasingly potent, efficient, safer, and able to circumvent cell insensitivity and drug resistance. Finally, this report provides exciting perspectives on the essential primary SARs of known dual Topo/G4 agents, and exploiting novel lead compounds featuring a dual mechanism at the telomeres, namely Topo inhibition/G-quadruplex stabilization, will help to open new avenues in drug design and development, resulting in more efficient drug candidates introduced onto the market and into the clinical pipeline.

## Figures and Tables

**Table 1:** Summary of the biological activities of FQA-CS and FQA-CR.

	FQA-CS	FQA-CR
G-4 interaction polymerase stop assay (IC_50_)	0.67 μM	0.06 μM
Topoisomerase II poison effects	+++	+
Cytotoxicity on MCF7 breast cells (IC_50_)	1.1 μM	0.46 μM
Major mechanism of action	Topo II poison	G-4 interaction

**Table 2:** Summary of the biological activities of indenoisoquinolines **3**–**11**.

Cpd	TopoI Inhibition ^1^	MYC Inhibitory Effect	G-Quadruplex Interaction K_d_ Values (nM) ^2^	MGM ^3^
3	+	strong	14.8 ± 0.3	0.24
4	+++	strong	N.D. ^4^	0.07
5	++	strong	N.D. ^4^	0.5
6	++	strong	8.5 ± 0.5	0.16
7	++	strong	7.3 ± 0.3	0.05
8	++++	strong	5.6 ± 0.2	0.06
9	++++	strong	23.9 ± 0.7	0.40
10	N.D. ^4^	weak	N.D.^4^	N.D. ^4^
11	0	no interaction	N.D.^4^	53

^1^ The relative topoisomerase I (TopoI)-inhibition levels of the compounds were previously determined and classified into six levels (0–5, +++++ = 5). ^2^ Apparent binding affinity K_d_ values (nM). N.D. indicates that the value was not determined due to the negligible change of fluorescence signal. ^3^ The MGM values for each compound are the average of GI_50_ values across the entire panel of NCI-60 cancer cell lines, where compounds with GI_50_ values that fall outside the test range of 10^−4^ to 10^−8^ M are assigned values of 10^−4^ or 10^−8^ M. ^4^ N.D. not determined.

**Table 3:** Summary of the biological activities of dibenzoquinoxalines **12**–**15**.

Cpd	TopoI Inhibition ^2^	G-4 Interaction/MYC Inhibition ^2^	IC_50_ (μM) ^1^
**12**	-	+++	1.9
**13**	+++	+++	1.1
**14**	+++	+++	0.7
**15**	+++	+++	1.1

^1^ IC_50_ is the half maximum concentration for cytotoxicity in TNBC cells at 24 h, determined using the CCK8 assay. ^2^ The c-MYC (or TopoI) inhibition levels were classified into four levels: inhibition (+++), medium inhibition (++), weak inhibition (+), and no inhibition (-).

**Table 4:** Summary of the biological activities of ruthenium(II) polypyridyl complexes **16**–**18** and cisplatin.

Cpd	Topos Inhibition	G-4 Interaction	HeLa	IC_50_ (μM) HepG_2_	A549
**16**	TopoI poison	Intercalation (stronger interaction)	37.45	46.31	52.45
**17**	TopoI/II poison	stacking mode	21.37	27.91	24.67
**18**	TopoI/II poison	stacking mode	23.85	21.90	24.06
cisplatin	-	-	16.75	12.15	18.55

## Data Availability

Not applicable.
